# Analysis of the “Sonar Hopf” Cochlea

**DOI:** 10.3390/s110605808

**Published:** 2011-05-30

**Authors:** Albert Kern, Stefan Martignoli, Wolfgang Mathis, Willi-Hans Steeb, Ralph Lukas Stoop, Ruedi Stoop

**Affiliations:** 1 Institute for Neuroinformatics ETHZ/UZH, Winterthurerstr. 190, 8057 Zürich, Switzerland; E-Mails: albert.kern@kzo.ch (A.K.); mstefan@ini.phys.ethz.ch (S.M.); stoopr@ee.ethz.ch (R.L.S.); 2 Theoretische Elektrotechnik, Leibniz Universität Hannover, Appelstr. 9A, 30167 Hannover, Germany; E-Mail: mathis@tet.uni-hannover.de (W.M.); 3 Applied Mathematics University of Johannesburg, 2006 Auckland Park, South Africa; E-Mail: whsteeb@uj.ac.za (W.-H.S.)

**Keywords:** artificial cochlea, biomorphic, mathematical analysis

## Abstract

The “Sonar Hopf” cochlea is a recently much advertised engineering design of an auditory sensor. We analyze this approach based on a recent description by its inventors Hamilton, Tapson, Rapson, Jin, and van Schaik, in which they exhibit the “Sonar Hopf” model, its analysis and the corresponding hardware in detail. We identify problems in the theoretical formulation of the model and critically examine the claimed coherence between the described model, the measurements from the implemented hardware, and biological data.

## 1. Introduction

The description of biological processes in terms of mathematics and physics is a challenge of great importance as an advanced step in explaining natural phenomena. Well worked-out examples connecting biology, nonlinear physics or mathematical bifurcation theory are relatively rare, implying that each new example is of great interest. The mammalian hearing system provides in this context an important example. The recent article “*Understanding the mathematics of hearing using electronic circuits*” by Hamilton, Tapson, Rapson, Jin, and van Schaik [1] therefore attracted our interest. The authors declare the aim of their presentation as follows: “*We show that the observed active behavior of the cochlea can be modeled as a change in the quality factor of the individual resonant sections in a discretized model, and that this has the dynamics which embody the Hopf bifurcation*”. Below, we discuss to which extent this aim is achieved. We discuss the mathematical terminology used in the approach, the appropriateness of the modeling (the “Sonar Hopf” approach), and finally the quality of agreement between the results from the model, from the electronic implementation, and biological example.

## 2. Fundamental Equation

In many applications, a differential equation $\frac{d}{dt}x=f(x)$ depends at least on one parameter λ, so that we need to study the solution manifold of a parametrized family of generally nonlinear differential equations. Since in such families generally different types of asymptotic solutions are present, it is of interest to study how these asymptotic solutions change their characteristics, upon a change of the parameter λ. The term “(local) bifurcation” is used to describe a “qualitative” or topological change of the solution behavior, upon a small (smooth) change of a system parameter λ (called the bifurcation parameter). At the bifurcation point itself, the system is at “criticality”. In most cases this means that as a function of the bifurcation parameter, the system is not differentiable at the bifurcation point, e.g., if the equilibrium point is about to become unstable and to give way to a stable oscillatory behavior. For continuous dynamical systems described by an ordinary differential equation (ODE), this can be indicated by a vanishing real part of one of the eigenvalues of the Jacobian, whereupon the ODE becomes nonhyperbolic at this parameter value. Among the different bifurcation types offering such a behavior is the Hopf bifurcation (see, e.g., [2]). Its normal form (*i.e.*, the simplest example displaying such a behavior) is usually written as [3,4]

(1) $$\frac{dz}{dt}=z(\lambda +b\;{\left|z\right|}^{2}),\;\lambda \;\in \;𝕉,\;b,\;z\;\in \;𝔺$$

This system admits a fixed point at (*x, y*) = (0, 0). If *Re*(*b*) < 0, a (supercritical) Hopf bifurcation occurs, leading to a limit cycle *z*(*t*) for *λ >* 0 with $r=\sqrt{-\lambda /\text{Re}(b)}$, *ω* = *Im*(*b*) and *z*(*t*) = *re^iωt^*. For *Re*(*b*) *>* 0, we have an unstable limit cycle for *λ <* 0. In this case, the bifurcation is called subcritical.

Rewriting the forced variant of this equation [3,4] erroneously, Hamilton *et al.* [1] start from

(2) $$\frac{dz}{dt}=\left(\mu +i\omega_{0}\right)z-z^{3}+{Fe}^{i\omega t},\;F,\;\mu ,\;\omega \;\in \;𝕉,\;z\;\in \;𝔺$$

From the cubic term, the authors conclude “*Hence, Equation (1) possesses important dynamics observed in the mammalian cochlea*”. As a first remark, we point out that because of the mixed product occurring in *z*^2^ ∈ 𝔺, the promised Hopf bifurcation will not occur. Explicitly, the bifurcation analysis of the system in the absence of drive yields

(3) $$\frac{d}{dt}\;\left(\begin{array}{c} x\\ y \end{array}\right)=\left(\begin{array}{l} \mu x-\omega_{0}y-x^{3}+3xy^{2}\\ \mu y+\omega_{0}x+y^{3}-3x^{2}y \end{array}\right)$$

This system has a fixed point *P* (0, 0) at the origin and two fixed points

(4) $$P_{2,3}=\pm \;\left(\begin{array}{c} \sqrt{-\mu +\sqrt{u^{2}+\omega_{0}^{2}}\;}(\mu +\sqrt{\mu^{2}+\omega_{0}^{2}})/(\sqrt{2}\omega_{0})\\ \sqrt{-\mu +\sqrt{\mu^{2}+\omega_{0}^{2}}}/\sqrt{2} \end{array}\right)$$

**Figure 1.**
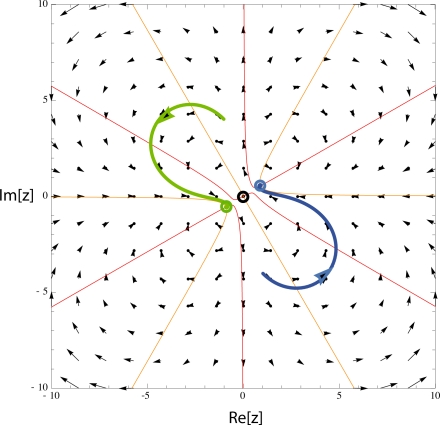
Vector field plot with isoclines (red lines) and fixed points (filled circles). Two solutions emanating from different initial conditions (green, blue) illustrate that a bifurcation from a central previously stable, fixed point to two stable fixed points has occurred (*ω*_0_ = 1, *μ* = 0.5, *F* = 0).

The first fixed point at the origin has the eigenvalues *λ*_*a*_1,2__ = *μ* ± *iω*_0_. Increasing *μ* from negative to positive values changes its nature from stable via elliptic to unstable. The eigenvalues of the two remaining fixed points are determined by $\lambda_{b,c}=-\frac{1}{2}(3\mu \;\pm \;\sqrt{\mu^{2}-15\omega_{0}^{2}})$. Upon increasing *μ*, their stability changes from unstable via elliptic to stable if *ω*_0_ ≥| *μ* | / $\sqrt{15}$ holds, which normally will be the case. A vector field plot of this system illustrates these results in Figure 1.

For observing the Hopf bifurcation, the term | *z*^2^ | *z*, instead of *z*^3^, is essential. This problem propagates through several works of the authors, leading to the conclusion that its origin cannot be accidental.

## 3. Bifurcation Terminology

The aim in the design of natural and artificial sensors is mainly the implementation of signal sensitivity, *i.e.*, the ability to react to faint stimuli with a greatly enhanced response in comparison to normal stimulation, and with damped response upon a strong stimulation. Behaviors of that sort are common in systems close to bifurcations [5–7]. The description of the “Sonar Hopf” model promotes the following view [1]: “*When operating at the bifurcation point* (*μ* = 0) *the system is said to be supercritically stable*”. Let us explain the origins, meanings and implications embodied in this description and point out why such a usage of terms should be abandoned. Locally, at the bifurcation point, the system is linearly marginally stable. According to the Hartman–Grobman Theorem, the behavior is then defined by higher order expansion terms. Although at the origin the normal form system’s behavior is characterized by asymptotic stability [2,8], a critical slowing down behavior towards this solution is observed: Transients evoked by small perturbations of the system relax generally but exorbitantly slowly. For the practical purpose in question, the system therefore reacts under these conditions in a manner that will be perceived as non-stable. If the subsystem describing the active part of the cochlea (in their language: the resonator) is at rest in the absence of a forcing, from where upon stimulation it is then led into stable oscillations, the transition is from a subcritical (below criticality) via the bifurcation point (criticality) to a supercritical state (where the system is now above criticality).

In addition to this local notion of bifurcation used, also a global notion exists. In the Hopf case, a bifurcation from a stable fixed point to a stable limit cycle of initially zero amplitude and fixed frequency is called “supercritical”. If we observe the collapse of an unstable limit cycle with a stable fixed point at the origin, by which the system is expelled to second, larger and stable limit cycle, this would be called “subcritical”. The second limit cycle also depends on the bifurcation parameter. Reducing the bifurcation parameter, the limit cycle generally becomes smaller, until it breaks down and the system falls on the stable solution at the origin, introducing hysteresis in this way. The problem in the terminology as used is that local and system level (*i.e.*, global) notions of bifurcations are combined on the same level without any warning.

“*When the system is supercritically stable and is driven at ω*_0_*, the response is nonlinear with high gain for small forcings and smaller gains for larger forcings*”. Exactly at the bifurcation point, the system is only linearly marginally stable and the gain *diverges* for ever smaller forcings [4]. Noise will naturally push the system beyond the bifurcation point, implying that the system then would be above criticality. The description continues “*If it is driven at* *ω* = *ω*_0_ + Δ*ω*, Δ*ω* ≠ 0, *then the response is approximately linear for sufficiently small input*”, where the obvious assumption is that we are still at the bifurcation point. This simple picture with respect to the detuning Δ*ω* only holds below criticality; for non-vanishing detuning on the supercritical side, additional frequencies will emerge with increased detunings. Together with phenomena of locking this substantially complicates the picture.

When the “Sonar Hopf” system is said to be “*supercritically operated*”, a local variation of the bifurcation parameter seems to be addressed but is referred to in terms reserved for the system level; systems displaying a subcritical Hopf bifurcation generally require huge changes in their system parameters (or are not capable at all) to produce supercritical Hopf bifurcations. A central statement made is “*Supercritical stability describes a system which is at the point of bifurcation from a single stable state to a bistable region, where the bifurcation is a Hopf bifurcation*” (see also [9]). However, a supercritical Hopf bifurcation does not change a steady state stable fixed point into a bistable solution, but a stable fixed point into a stable time varying oscillation (a limit cycle). In the local picture no bistability is involved. A system able to display a supercritical Hopf bifurcation can always be operated below or above—or more difficult, at—the bifurcation point. The important information whether the “Sonar Hopf” system is operated below (“subcritical”) or above (“supercritical”) criticality remains unclear, within the description given. Whereas a system close to a bifurcation point could be used on either side of the bifurcation point as a small signal amplifier [5–7], it should be also noted that each bifurcation and each side with respect to the bifurcation point brings about its own fingerprint (e.g., how the nonlinearity scales [10]). It is only upon a close inspection of the design of their device [9] that the reader arrives at the guess that the “Sonar Hopf” device is in fact designed to operate *below* criticality.

## 4. “Sonar Hopf” Equation

Even more importantly, a genuine Hopf bifurcation might not necessarily be involved in the described “Sonar Hopf” cochlea framework. In the approach, a feedback system is used to vary the quality factor of the basilar membrane at each the intensity (and frequency) of the input signal [Note: In contrast to how the authors of [1] perceive it, we feel that not the damping by the basilar membrane, but the viscous damping of the fluid much is the governing biophysically element for the dissipation active at a given point along the cochlea]. To change the quality factor *Q* ∈ 𝕉, the quantity

$$\zeta :=\frac{1+AQ}{Q}$$

where *A* ∈ 𝕉 is the feedback gain term, is monitored. For the resonant system, the equation

(5) $$\Gamma =(\frac{\omega_{0}s}{s^{2}+s\omega_{0}\zeta +\omega_{0}^{2}})\;(\Theta +A\Gamma )$$

is obtained. The claim then is that “*A feedback loop that adds the weighted energy of its output signal to its input possesses the dynamical properties of a Hopf bifurcation*”.

From a general discussion point of view, it is true that by means of an appropriately operated feedback loop, a large class of nonlinear dynamical systems can be brought from fixed-point into oscillatory behavior—some of them by means of a Hopf bifurcation, but not necessarily—as well as from chaos into regular oscillations, and so on [11–13]. Whether the feedback mechanism will produce the desired oscillation and what kind of bifurcation it will demonstrate depends on the delay necessarily involved, and the feedback strength. Moreover, every control system when active has effects additional to the original system, compare, e.g., [14,15]. In full generality, the above claim therefore is incorrect.

More specifically, the “Sonar Hopf cochlea” extends a resonator model by positive feedback, with the objective to use a parametrized positive feedback function so that a limit cycle emerges by means of the Andronov–Hopf mechanism. Unfortunately the discussion of the Andronov–Hopf bifurcation in feedback systems as provided is fragmentary, partly wrong and historically inadequate. The key point made in [1] is that the equation

(6) $$\frac{1}{\omega_{0}}\frac{d\Gamma }{dt}+(\zeta -g\mu )\Gamma +\int \omega_{0}\Gamma dt+g\Gamma {\left|\Gamma \right|}^{2}-\Theta =0$$

precisely models their electronic system. About this equation the authors say “*.. is in the form of a first order system that displays the dynamical properties of the Hopf bifurcation.” “We are on the critical point, and hence supercritically stable when ξ = gμ. Therefore, using this model we are able to tune the resonators to stable, critical, and unstable operating points simply by varying μ.*” From the biophysical point of view, the introduction of the feedback function

(7) $$A=g(\mu -{\left|\Gamma \right|}^{2})$$

that leads to this equation seems to be motivated by the nonlinear term of the Poincaré’s Hopf equation normal form, adequate for systems of the van der Pol equation type. Equation (6) is, however, not of the van der Pol type. Therefore, the choice (7) is arbitrary, since it is unrelated to any modeling assumptions of the cochlea. Also authors’ conclusion that a Andronov–Hopf bifurcation arises is in no way obvious. We note that in contrast to the statement provided in [1], the fundamental integro-differential Equation (6) is of second and not of first order. A first order system would not be able to generate stable oscillations.

The analysis provided up to this point does not reach beyond a steady state, pure tone analysis. If a feedback mechanism is needed to keep it at the desired bifurcation location, even if the amplification had the correct form, this would not be instantaneous. Different unwanted effects all known from delay control mechanisms must be expected. The dynamic response, a very essential feature of a cochlear device, cannot safely be retrieved from this oversimplified picture. Whereas the ultimate mathematically precise description would probably have to be given in terms of spectra of resonances [16], an “acceptably correct” mathematical description would at least require the framework of delay differential equations. The proof for the occurrence of a Hopf bifurcation given in the manuscript [17] unfortunately points to the void. The presented framework is therefore insufficient for pinning down auditory Hopf amplification behavior by approximatively implementing compressive nonlinearity (to whatever extent of precision, see the discussion below). The characteristics of a Hopf system embrace much more than that, and the mammalian hearing process makes, in fact, heavy use of many of these properties [18].

**Figure 2.**
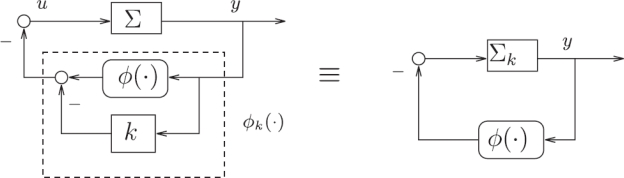
Equivalent representations of the Lure SISO nonlinear system described.

**Figure 3.**
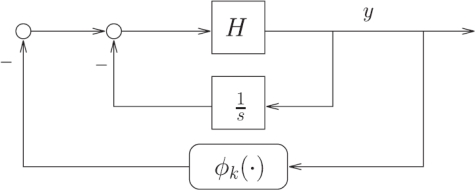
Forcing the Hopf bifurcation with an integrator in the feedback loop and *H* passive. The case *H*(*s*) = 1*/s* corresponds to Lienard systems.

From an engineering point of view, ignoring relevant mathematic details in this description, the “Sonar Hopf” construct is an example of well-known feedback systems with limit cycles, known under the name of Lure systems. For a systematic engineering approach to Lure type systems with nonlinear feedback and limit cycles, the theory of Sepulchre and Stan [19] would have been helpful (see also the dissertation of Stan [20] for further details). This analysis can be applied to rather general linear systems (including resonators) with nonlinear feedback functions, resulting in a sophisticated formulation of the Andronov–Hopf bifurcation of Lure systems. Within this theory [19], a sound mathematical discussion of the system class depicted in Figure 4 of [1] could be given, along the Figures 2 and 3 as follows.

The feedback system of Figure 3 has the following energy interpretation: Passivity of H_k*_ (with k* identifying the bifurcation point) allows for a lossless exchange of energy between two storage elements. The static nonlinearity *ϕ_k_* “regulates” the dissipation in the feedback system, restoring energy when it is too low, and dissipating energy when it is too high. In the celebrated van der Pol oscillator, the two storage elements are a capacitor and an inductor, whereas the dissipation is regulated by means of (for instance) a tunnel-diode circuit modeled as a static negative (*i.e.*, active) resistance. Theorem 2 in [19] extends this feedback mechanism for oscillations to higher-dimensional systems. It can be noted that the local argument given there in the proof of Theorem 2 essentially shows that the (arbitrary) passive system *H* reduces to an integrator on the center-unstable manifold. It should also be noted that, starting from an arbitrary passive system *H*, putting an integrator in the feedback loop as in Figure 3 forces the Hopf bifurcation scenario because of the resulting presence of a zero at *s* = 0 in the transfer function

(8) $$G_{k}\;(s)=\frac{sH(s)}{s+(1-k)sH(s)}$$

We feel that for a “scholarly” approach as taken in the paper, these relations fundamental for the understanding of the “Sonar Hopf” concept should not be ignored. We also note that the electronic literature notion of an “*operating point*” generally does not embrace the unstable fixed point at the origin. Finally, the absolute signs used in Equation (6) make no sense in the given context because this equation is an equation in the reals.

## 5. “Sonar Hopf” Electronic Cochlea

**Figure 4.**
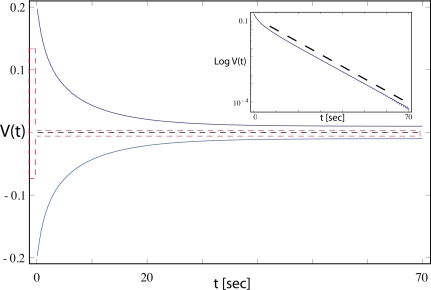
Blue: Envelope of the exponential decay from larger towards smaller stimulation according to Equation (5), using the same temporal units as in [1]. Red: Results from the device (according to Figure 5(b) in [1]). The asymmetric response and the substantial decay mismatch indicate that Equation (5) does not provide an accurate description of the electronic device.

**Figure 5.**
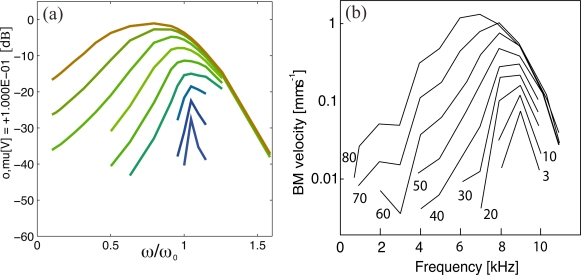
(a) Original measurements (2004) from a cochlea with directly implemented Hopf amplifier outer hair cells, reproduced in [21]. Input stimulation range: 80 dB. For more refined measurements and more complete information see, e.g., [22,23] and, most recently, [24]. (b) Chinchilla cochlea [25]. To be compared with Figure 6(a) from [1].

The main claim then is that the response of the constructed electronic device is faithfully represented by Equation (5). As a proof, in Figure 5 of [1], two device measurements are shown. Of interest is that in the response provided in Figure 5(b), even the most fundamental traces of a Hopf bifurcation are missing. Close to the bifurcation point, a Hopf system will display the “critical slowing down” phenomenon (in *this* context, a citation of [17] would have been helpful). Moreover, there is a huge offset from the zero line, pointing into different directions depending on the signal size. A proper Hopf system would be hardly doing that. To corroborate these objections, we numerically simulate their equation, where we use Equation (4) in [9] at the parameters given therein (note that in this equation, the absolute signs make no sense either). Using an effective *μ_eff_* := *μg* − *R* = −0.2, corresponding to the third curve displayed in Figure 2 of [9], we find a critical slowing down that contradicts the behavior of their device, see Figure 4. Therefore, the behavior of their electronic system is not generated by a genuine Hopf system.

The concluding statement “*We present a two dimensional (2D) model of the cochlea which includes supercritically stable amplification (a Hopf bifurcation) in a physiologically realistic way*” seems therefore insufficiently supported by mathematical and biological evidence. The implementation of a compressive nonlinearity similar to that observed in a Hopf system is not sufficient for establishing a Hopf system. The cochlea active amplifiers are located in the outer hair cells that are likely to undergo Hopf bifurcations. From here to arrive at the postulated “*change of the quality factor*” in a “physiologically realistic way” [1] seems to require more care, even more so if a “biophysically realistic” meaning were implied as well—perhaps not necessarily from an electronic device design point of view, but certainly from a biophysics and mathematics point of view.

For the reader it would also have been interesting to be able to compare the “Sonar Hopf” implementation against a direct implementation of active Hopf hair cells amplifiers, such as the earlier electronic Hopf cochlea [21,26,27], which is based directly on a Hopf process of the auditory outer hair cells.

Recently, this device was used to gain insight into the perception of mammalian hearing [18]. For this to substantialize, extending the earlier presentation [22], in [18] a generic comparison between this cochlea and the biological measurements was provided [24]. These results summarize the state of art of the correspondence between electronic Hopf cochlea designs and the mammalian hearing system that has already been achieved. We feel that the “Sonar Hopf” cochlea article could have benefitted from bringing these results (known to the authors) to the attention of the reader.

## 6. Conclusion

Our analysis shows that the close correspondence between the “Sonar Hopf” device and behavior of a genuine system at a Hopf bifurcation point claimed in [1] is mostly formal. In essential parts, the biological foundation is not from first principles and the mathematical formulation is misleading. With our review, we hope we have identified and clarified the most pertinent shortcomings and have pointed out contexts that might have been helpful for the reader to better understand and assess the “Sonar Hopf” cochlea concept.
